# The effect of a social network-based cognitive behavioral therapy intervention on the severity of premenstrual syndrome symptoms: a protocol of a randomized clinical trial study

**DOI:** 10.1186/s13063-022-06290-0

**Published:** 2022-04-23

**Authors:** Zainab Alimoradi, Somayeh Rajabalipour, Khaled Rahmani, Amir H. Pakpour

**Affiliations:** 1 Social Determinants of Health Research Center,Research Institute for Prevention of Non-Communicable Diseases, Qazvin University of Medical Sciences, Qazvin, 34197-59811 Iran; 2 Student Research Committee, Qazvin University of Medical Sciences, Qazvin, Iran; 3 Social Determinants of Health Research Center, Kurdistan University of Medical Sciences, Sanandaj, Iran; 4 Department of Nursing, School of Health and Welfare, Jönköping University, Jönköping, Sweden

**Keywords:** Premenstrual syndrome, Cognitive behavioral therapy, Theory-based intervention, Social network-based intervention, Self-efficacy

## Abstract

**Background:**

Premenstrual syndrome (PMS) is one of the most widespread menstrual disorders in women of reproductive age. This recurrent syndrome is a combination of physically, mentally, or behaviorally disturbing changes occurring during the secretory phase of the menstrual cycle. The aim of this study is to determine the effect of a cognitive-behavioral therapy-based educational intervention using social networks on PMS in female health center employees in Rudbar, Iran.

**Methods/design:**

A randomized superiority controlled trial will be conducted involving 140 female employees of health centers affiliated with the Guilan University of Medical Sciences, Rudbar. The study involves a block size of 4 and 6 in a randomly varied order, 140 women who meet all the inclusion criteria will be randomly and equally divided into 2 groups: the intervention and the control groups. Those in the former group will receive a cognitive-behavioral therapy-based treatment for eight consecutive weeks on the social network platform WhatsApp; however, those in the control group will not be offered any treatment except usual care practices (unprotocolized usual care). The study’s primary outcome is the severity of PMS symptoms, and the secondary outcomes include general self-efficacy, work-related quality of life, the impact of PMS on daily life, coping with the symptoms, and experiencing anxiety and depression at the beginning of the study to identify people with PMS. A daily record of the symptoms will be completed for two consecutive months by all female employees aged 20–45 years who wish to participate in the study. According to the initial screening, those with moderate to severe PMS will be included. We will use the MLwin software for the analyses. All questionnaires will be completed by both groups immediately and 8 weeks after the termination of the treatment. The data will be analyzed using linear mixed-effects modeling with random intercepts and slopes.

**Discussion:**

It is anticipated that the findings of the present study may demonstrate the effectiveness of the cognitive behavioral therapy intervention on the severity of PMS symptoms that could guide healthcare providers in opting for suitable treatment alternatives for the syndrome.

**Ethics and dissemination:**

The research proposal is approved by the Human Ethics Committee of Qazvin University of Medical Sciences (IR.QUMS.REC.1399.252). The results of this intervention trial will be submitted for publication in a peer-reviewed research journal.

**Trial registration:**

Iranian Registry of Clinical Trials IRCT20180218038789N4. Registered prospectively on October 28, 2020

## Introduction

Premenstrual syndrome (PMS) is one of the most common menstrual disorders in women of reproductive age [1]. This syndrome is a recurrent combination of bothersome physical, psychological, or behavioral changes during the secretory phase of the menstrual cycle [2] which decrease rapidly with the onset of menstruation [3, 4]. Its most common symptoms are anxiety, depression, fatigue, anger, irritability, feeling out of control, confusion, changes in appetite and sleep, bloating, and breast tenderness. These symptoms and their severity unquestionably vary among females, rendering its diagnosis difficult [5–7].

The high prevalence of PMS is a major concern in many settings [8]; however, due to the variety and severity of its symptoms, it is challenging to estimate its prevalence [9]. Yonkers et al. [10] and Potter et al. [11] have estimated that PMS affects 20% of women of childbearing age.

The PMS symptoms affect women’s quality of life and disrupt many aspects of it, including daily activities, interpersonal relationships, social activities, sexual function, and occupational function. Additionally, they can cause disturbances in couples’ relationships, incompatibilities with children, absence from workplaces, and social consequences such as increased accidents and abuse [12]. The surge in job conflicts as well as the growing demand for divorce by women in the premenstrual period has led to confusion and serious dangers in their lives [13, 14]. The syndrome escalates the direct costs of treatment and also the indirect expenses associated with reduced work productivity and efficiency to a significant extent [15].

Therapeutic goals for treating PMS include reducing the extent and severity of symptoms as well as their impact on personal activities and relationships and improving the quality of life [16]. Because the underlying cause of the condition is unknown, several treatments have been recommended to regulate it, including medication, non-pharmacological treatment, and surgical treatment [17]. According to the American College of Obstetricians and Gynecologists [18], the choice of treatment strategy depends on the severity of the PMS symptoms. The use of non-pharmacological methods is usually employed as the first strategy for mild to moderate cases, and drug remedies are used for those that are severe or interfere with daily functioning [19]. However, a cross-sectional study showed that only 48% of the participants with PMS sought medical treatment [20].

Pharmacological treatment of PMS includes vitex agnus-castus (only herbal medicine proven to control PMS-associated mood swings and irritability), combination oral contraceptives, and selective serotonin reuptake inhibitors (considered as the first-line treatment of PMS with predominantly emotional symptoms) [17]. Because drugs are often associated with significant side effects such as nausea, weakness, and headache, a substantial number of women discontinue their use despite the evidence of their effectiveness. Side effects, and consequently, the high rate of non-adherence as well as the desire of women to cope with the symptoms naturally, have undoubtedly limited the practicality and applicability of medications [21–23]. Concerns regarding their aftereffects have led those with PMS to request non-pharmacological interventions [24].

Non-pharmacological treatment includes lifestyle modification, dietary supplementation, and cognitive behavioral therapy [17]. Nonetheless, there is limited evidence to support the effectiveness of lifestyle modifications [25–28] and dietary supplementation [29–31] as independent forms of treatment of PMS. Although the causes of PMS are multidimensional and consist of physical, psychological, and cultural factors, current studies have mainly focused on physical interventions. Psychological interventions have been suggested as another treatment alternative for PMS symptoms; CBT is a psychological approach with the best evidence of its usefulness [32, 33].

CBT is based on the idea of how we think (cognition) and feel (emotions) and the manner in which we interact with each other (behavior). Therefore, negative and irrational thoughts can cause discomforts and problems [34]. CBT consists of limited weekly sessions focusing on correcting negative and abnormal thoughts as well as educating individuals on effective adaptive mechanisms. Its effects are achieved over time and, last, even when the sessions are completed. Conversely, drug therapy has a very rapid influence that ends with its discontinuation [35, 36]. These outcomes demonstrate the potential of CBT as a promising treatment alternative leading to long-term sustainable progress and also highlight the important role of coping strategies. However, these findings require further research [37, 38].

Due to the serious consequences and negative effects of this syndrome on the activity and performance of individuals, the very important role of women in the family and society, increasing the number of workers, and very few studies on the effects of PMS in the workplace, the target group of this study was health workers. They are one of the most important occupational groups in promoting community health and are themselves role models for different groups of people. Therefore, the present study was designed to determine the effect of a CBT-based educational intervention using social networks on PMS in female employees of health centers in Rudbar, Iran.

## Methods

### Study design

**Fig. 1:**
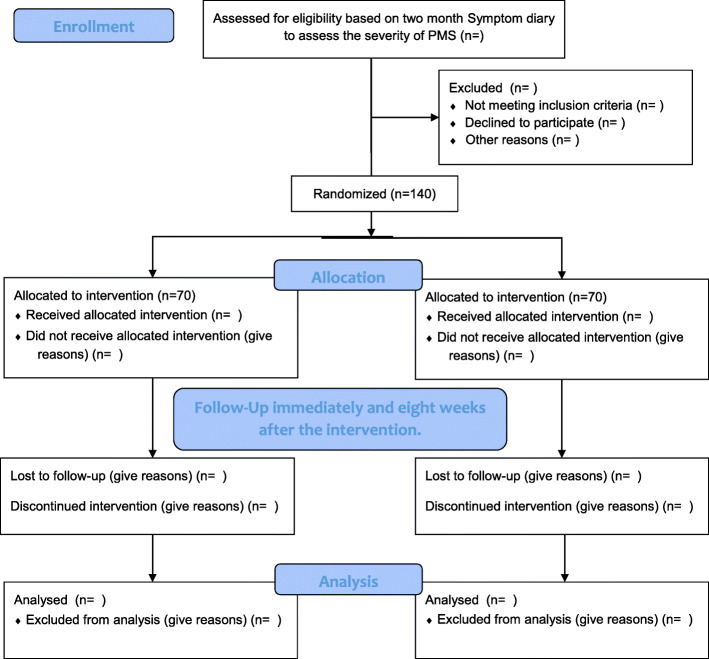
CONSORT flow diagram

The present research is a block randomized superiority controlled trial that will be performed in the health centers affiliated with Guilan University of Medical Sciences, Rudbar, Iran. Figure 1 shows the study CONSORT flowchart.

### Participants

The research population in the present study includes female employees aged 20–45 years in the Rudbar health centers who will be invited to participate using the convenience sampling method.

#### Inclusion criteria

The following are the inclusion criteria:

- Willingness to participate in the study
- Age 20–45 years
- Moderate-to-severe PMS or PMDD (based on PMS screening using a daily symptom registration form)
- Having internet access
- Possessing a smartphone

#### Exclusion criteria

The following are the exclusion criteria:

- Diagnosis of psychosis or bipolar disorder, ED, and moderate-to-severe depression
- Participated in a psychotherapy program for PMS at present or in the past
- Acute suicide tendencies
- Childbirth or breastfeeding during the past three months
- Pregnancy
- Gynecological diseases (hysterectomy, oophorectomy, cervical cancer, polycystic ovary syndrome, endometriosis, and infertility)
- Using any kind of antidepressants, benzodiazepines, antipsychotics, oral contraceptives, or hormones over the past 3 months

### Sample size estimation

The intervention effect will be evaluated by comparing the mean score of PMS symptom severity (primary outcome measure) between the two groups. Thus, the sample size was calculated based on the ability to detect a clinically relevant difference of 30% between the two groups in 8 weeks after intervention (30% reduction in the mean PMS score). This sample size accounted for a type I error rate of 0.05, any-pair power of 0.95, and all-pair power of 0.85. Based on the evidence from a previous study [39] and assuming a mean = 163.6, SD = 89.5, in group 1 and mean = 115.0, SD = 89.5, in group2 and an estimated reduction of 30% in the mean PMS score due to the intervention, the target sample size was calculated at 62 patients per group, with a 10% loss to follow-up, and to increase the power of the study, we selected 70 women in each group for a total sample size 140.

### Sampling and randomization

First, all female employees of the health centers will be invited to participate in the study using an announcement. Second, women will be screened for having moderate-to-severe PMS. Third, after a run-in period, primary evaluation of study subjects, and obtaining written informed consent, women who had our eligibility criteria will be randomly assigned to two groups (intervention and control) by using the permuted random blocking method with four and six blocks. Considering that two groups will be studied, four and six blocks in a randomly varied order are utilized and each group is assigned a letter (A: intervention group; B: control group). Since we have a list of all eligible women, the formation of permuted blocks and random allocation of individuals to two groups of intervention and control will be determined prior to the initiation of the research. Indeed, block size of 4 and 6 in a randomly varied order is used to conceal the allocation sequence.

In order to hide the allocation sequence, the type of intervention is written on the cards based on it, and placed in envelopes in a matte package numbered from 1 to 140. In this case, the person who receives intervention code 1 completes a questionnaire with the same code. The assignment of participants to their allocations will be done by an independent healthcare provider. Due to the nature of the study intervention, modification of random allocation during the study and blinding of study participants and outcome evaluators is almost impossible. Although the content of the intervention will be delivered online using the social media platform and blinding is difficult, we tried to maintain the confidentiality of data through coding of study subjects and not asking their identity.

### Intervention

The content of the intervention will be based on the principles of CBT and will be delivered online using the social media platform WhatsApp (Table 1). The intervention lasts for eight consecutive weeks, and its components are divided into 14 individual sections. It commences with an introductory section and ends with one on relapse prevention. The rest of the sections are divided into two categories: cognitive strategies and suggestions for behavioral lifestyle changes. Consequently, except for the initial and the final week, participants work on two parallel sections each week. For these sections, an approximate working time of 5 h per week is recommended.

**Table 1:**
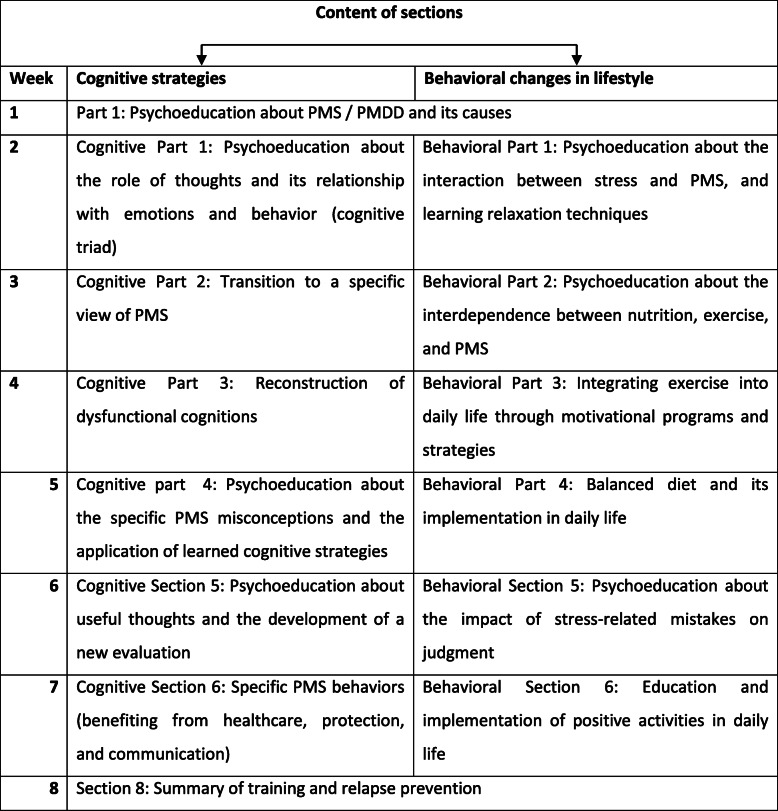
Contents of the CBT-based treatment for the intervention group

The first part includes psychoeducation about PMS/PMDD, its causes, and treatment. The cognitive strategy section provides information and strategies for identifying and correcting ineffective thoughts, especially PMS-specific cognitions, misbehaviors, and misconceptions. The sections cover suggestions for lifestyle changes, such as stress reduction, exercise, and a balanced diet. In the final section, a summary is provided, along with a plan to sustain the benefits and prevent relapse. All sections include practical exercises for applying and practicing the theoretical content. Additional audio files are provided to facilitate the performance of these exercises, such as an advanced muscle relaxation training program or an audio instructional exercise to gather thoughts related to PMS. Textual information and audio files are delivered through social networks. Weekly feedback on the intervention is obtained to assess the participants’ compliance with the intervention. If they do not submit their feedback, they will receive two reminders.

The following is the primary outcome:

- Severity of PMS symptoms

The following are the secondary outcomes:

- General self-efficacy
- Quality of working life
- The effect of PMS on daily life
- Coping with premenstrual symptoms
- Anxiety and depression

### Data collection tools

A self-reported measure will be used for the purpose of data collection. Data will be gathered using a multi-part questionnaire that includes the following sections.

#### Demographic information

*Demographic information* will be examined using items such as age, marital status, level of education, height, weight, number of children, work experience, and type of employment.

#### Symptom diary to assess the severity of PMS

The severity of premenstrual symptoms was assessed based on the severity of 27 physical, and behavioral symptoms as well as 3 items related to interference with social activities were measured using a PMS symptom diary validated by Janda et al. [40] prospectively. Therefore, the participants will be requested to complete a daily prospective record of their symptoms during two consecutive menstrual periods using a 4-point Likert scale (0 = *normal*, 1 = *low*, 2 = *moderate*, 3 = *severe*). The effects of these symptoms on social aspects are examined. According to the DSM-V criteria for severe PMDD and PMS, a minimum of 5 symptoms (including a behavioral symptom) of the 27 primary items should be present (more than 2), with at least one of them related to the impact on social aspects (greater than 2) for no less than 2 days in the luteal phase [40].

For each woman, scores related to symptom severity (SI score) and severity of the interference with social activities (INT score) will be calculated. The first 27 items are used to calculate the SI score, and items 28 to 30 are used to calculate the INT score, which indicates social and occupational interference. These scores are calculated for the luteal phase. Luteal phase scores are calculated according to the last 7 days before menstruation. Only symptoms that are rated higher than or equal to 2 for at least 2 days will be entered into the calculation. The values of all items with a score greater than or equal to 2 for at least 2 days are added together and divided by 7 [40]. Therefore, the average severity of PMS symptoms per person will be calculated.

It should be noted that the symptom record sheet should be completed 2 months before the intervention starts, in order to diagnose PMS symptoms and assess the severity of PMS; the average of these 2 months will be considered as a baseline score. The severity of PMS will be assessed for 1 month immediately and 8 weeks after the end of the intervention to evaluate the short-term and long-term effects of the intervention.

#### Impact on everyday life and symptom intensity

This questionnaire was designed by Kues et al. in 2015. Its 18 items measure the impact of PMS on daily life through two subscales of psychological and functional effects. The validity of the measure was confirmed using exploratory factor analysis. Cronbach’s alpha coefficient of 0.9 for both subscales showed an acceptable level of reliability [41]. In the present research, this questionnaire has been translated into Persian, and its validity and reliability will be examined before the main study. This questionnaire will be completed before, immediately, and 8 weeks after the intervention.

#### Anxiety and depression

In the present study, the Hospital Anxiety and Depression Scale (HADS) will be used to assess anxiety and depression. This instrument was developed in 1983 by Zigmond and Snaith to screen for anxiety disorders and depression in non-psychiatric clinic patients. This scale comprises 14 questions in 2 subscales, namely, anxiety and depression. Each item is scored on a 4-point Likert scale ranging from 0 to 3. The maximum score that can be obtained for each subscale is 21. Scores above 11 on each subscale are considered abnormal, scores from 8 to 10 indicate borderline cases, and those from 0 to 7 are deemed as normal [42]. Bjelland et al. examined the psychometric properties of the HADS in a systematic review and concluded that it was an appropriate tool for assessing anxiety disorders and depression for different groups, including somatic patients, psychiatric patients, primary care clients, and the general population [43]. The psychometric properties of the Persian version were reviewed and confirmed [44]. This questionnaire will be completed before, immediately, and 8 weeks after the intervention.

#### Coping with premenstrual symptoms

The Premenstrual Change Coping Inventory is a self-report 17-item scale that measures individual strategies to deal with PMS. It includes three subscales, “seeking positive affect-inducing activities,” “seeking support,” and “healthcare use behavior” that are scored on a 4-point Likert scale from 1 (*not at all true*) to 4 (*totally true*). A higher score indicates a better status of coping with PMS [45]. In the present research, this questionnaire has been translated into Persian, and its validity and reliability will be assessed prior to the main study. This questionnaire will be completed before, immediately, and 8 weeks after the intervention.

#### The Work-Related Quality of Life Scale

The Work-Related Quality of Life Scale was first developed in England to assess the quality of occupational life in health care workers. Its validity and reliability were verified among medical personnel in the UK [46]. In the present research, this questionnaire has been translated into Persian, and its validity and reliability will be assessed prior to the main study. This questionnaire will be completed before, immediately, and 8 weeks after the intervention.

#### The General Self-Efficacy Scale

The General Self-Efficacy Scale was designed by Schwarzer and Jerusalem in 1995 to assess a person’s belief in their ability to respond to new or difficult situations and to deal with any obstacles and related problems. It consists of 10 items that are answered on a 4-point Likert scale ranging from “not at all correct” to “exactly correct.” Each person’s score is calculated as the average of the responses to all the questions [47]. A cross-cultural validation study using samples from 25 countries showed that this scale is unidimensional, having a Cronbach’s alpha of 0.75–0.91 [48]. This questionnaire will be completed before, immediately, and 8 weeks after the intervention.

### Timing of the outcome assessment

**Fig. 2:**
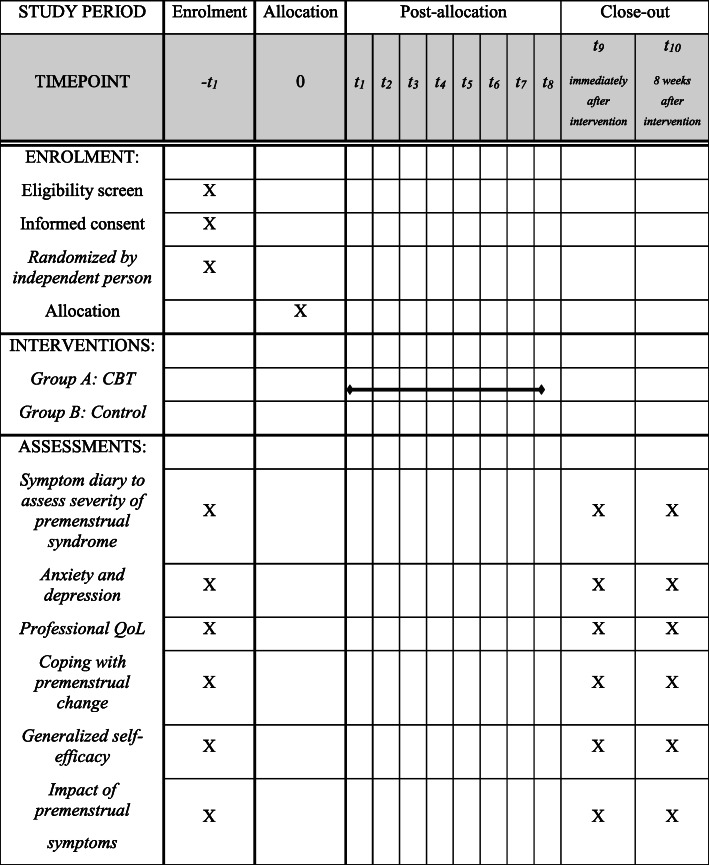
Schedule of enrollment, interventions, and assessments

All outcome variables will be assessed before, immediately, and 7 weeks after the intervention (Fig. 2).

### Intervention fidelity

The second author is responsible for sampling and performing the intervention. However, the research group (specifically the last author) will supervise all stages of research implementation and content preparation.

### Data management

In this study, the data obtained from the questionnaires will be coded and entered into the SPSS software version 23. It should be noted that baseline data of all women identified as satisfying the eligibility criteria would be recorded initially. Data of discontinuing cases will be compared with the same data from those who agree to remain in the study. This will allow determining the representativeness of study subjects and generalizability of the finding. As mentioned above, this is an intervention study, and we did not find any adverse events for CBT in the literature; however, researchers plan is to report any unforeseen events or incidents during the study.

### Data analysis method

The effects of the intervention will be analyzed using a two-level linear mixed model in which repeated assessments will be time (variables from the beginning of the study to the end of the intervention and 3 months after its completion) at the first level and female employee at the second level. The model will be adjusted for the impact of potentially confounding variables (e.g., age, work experience). Mixed models are commonly used to handle missing data. Therefore, no additional steps will be taken to manage the missing data. Restricted iterative generalized least square estimation will be performed to produce unbiased estimates of the random parameters. The data will be analyzed by the intention to treat with the MLwiN 2.27 software.

### Ethical considerations

The research protocol has been reviewed and approved by the Ethics and Human Research Committee of the Qazvin University of Medical Sciences (decree code: IR.QUMS.REC.1399.252). It is registered at the Iranian Clinical Trial Registration Center under the decree code IRCT20180218038789N4. In addition, the following ethical considerations will be taken into account throughout this study: obtaining written informed consent, voluntary nature of participation, ability to withdraw from the research at any time, and confidentiality of information even during the publication of the findings. All ethical issues related to clinical trials will be considered. Moreover, informed consent during the recruitment phase will be obtained. CBT is a defined intervention protocol that has been used in many previous studies and is almost unchanging. It should be noted any change in protocol or its implementation will be informed to the main stakeholders of the study including the study subjects, trial team, and research deputy of Qazvin University of Medical Sciences. Ethical committee affiliated to the Qazvin University of Medical Science will select one independent expert as an independent trial audit. This person will monitor all steps of conducting the research including data.

## Discussion

PMS is one of the most common menstrual disorders in women of reproductive age [1] that requires interventions to reduce its severity and symptoms, as well as decrease their impact on activities and personal relationships and, in general, improve the quality of life [16]. CBT is a psychological intervention that has the best evidence of its effectiveness [32, 33]. Although the results of the currently limited research have found it to be a promising treatment option with long-term sustainable outcomes, these findings still require further research [37, 38]. To the best of our knowledge, the present research is innovative in several respects.

First, in previous studies, the target group of healthcare providers was not taken into consideration. Previous studies have shown positive results regarding the effect of online CBT interventions for reducing functional and psychological dysfunction caused by PMS, its impact on daily life, the severity of symptoms, and the related disability in different groups including urban women [49], rural women [50], and students [51, 52]. However, according to the research group, most studies were not theory-based and did not use behavior modification techniques. Therefore, in the present study, an attempt was made to design an intervention program using the framework of self-efficacy theory to minimize the limitations of previous research.

Second, designing an intervention program within the framework of self-efficacy theory is an attempt to minimize the limitations of previous studies. Perceived self-efficacy is a judgment of an individual’s ability to perform a particular set of actions. It is developed through skillful practice, substitution learning, verbal persuasion, and physical responses to specific situations. Overestimation of capabilities can lead to failure, while underestimation can reduce challenges and improve capabilities. It is closely related to perceived health control and the belief that a person has the skills to perform certain functions that actually affect their health level. Confidence in a person’s abilities to perform these tasks can increase the likelihood of attempting them. In addition, self-efficacy is a significant component of hard work and the capacity to withstand stress and change [53]. Some studies have shown that PMS onset and progression are related to stress [54, 55]. However, premenstrual physical and hormonal variations have profound effects on women’s lives, which when combined with stress, exacerbate its negative impacts [56]. Nevertheless, stress is at the forefront of physical and psychological health factors. It is closely associated with PMS as it increases its symptoms in physical, behavioral, and emotional dimensions [57]. It is a consequence of a person’s psychological assessments of the situation [58]. It appears that this syndrome is more predominant and severe in educated women (same as the selected target group of the current study), which could be due to increased stress experienced by them [59].

Third, CBT will be provided via an Internet platform. Internet-based CBT is readily available and has several benefits [60–62]. For example, treatment can be offered to many women regardless of their geographical location, and it can be accessed by the patient and the therapist at any time, thus requiring no prior appointment. An important advantage of such Internet-based methods is that most of them provide confidentiality that can reduce barriers to helping women who are scared or have previously experienced stigma due to PMS, PMDD, or seeking psychotherapy [37, 63]. The value of health education programs depends on the accurate use of the related theories and models. In other words, the existence of appropriate theoretical support along with basic health needs will increase the effectiveness of these programs [64, 65]. Self-efficacy refers to the confidence that an individual has in their ability to pursue a behavior. It plays a pivotal role in behavior modification [66]. Bandura cites self-efficacy as the most important determinant of behavior change, as it can influence a person’s choice of actions. Furthermore, it makes them put additional effort into achieving the action and enduring obstacles [67].

Additionally, the use of a randomized controlled trial design with a concurrent control group and significant sample size are other strengths of this study. It is expected that these findings may demonstrate the effectiveness of the CBT intervention on the severity of PMS symptoms that could guide healthcare providers in selecting appropriate treatment alternatives for this condition.

### Trial status

The recruitment has not yet begun; however, all necessary permissions have been acquired; the estimated date of recruitment is December 20, 2020. The expected date for completing the recruitment is February 30, 2021.

## Data Availability

Not applicable as this is a protocol.

## Abbreviations

PMS: Premenstrual syndrome
PMDD: Premenstrual dysphoric disorder
CBT: Cognitive behavioral therapy
COVID-19: Coronavirus disease
HADS: Hospital Anxiety and Depression Scale
